# Caroli Syndrome: Challenges in Early Diagnosis for Infants

**DOI:** 10.7759/cureus.80784

**Published:** 2025-03-18

**Authors:** Olfa Asbik, Amal Hamami, Abdeladim Babakhouya, Maria Rkain

**Affiliations:** 1 Department of Pediatrics, Mohammed VI University Hospital, Faculty of Medicine and Pharmacy, Mohammed I University of Oujda, Oujda, MAR

**Keywords:** caroli syndrome, early diagnosis, infant, intrahepatic bile duct dilatation, liver

## Abstract

Caroli syndrome is an uncommon disorder characterized by congenital, segmental dilation of the intrahepatic bile ducts, often widespread and accompanied by liver fibrosis, progressing to juvenile portal hypertension. Although present at birth, this congenital anomaly is often undetected until adulthood. The diagnosis is usually delayed due to its clinical latency. In this case, a one-year-and-eight-month-old infant with abdominal distension and hepatosplenomegaly underwent imaging, which revealed segmental dilation of the intrahepatic bile ducts. Early detection allowed for timely management, reducing the risk of severe complications. Early diagnosis of Caroli syndrome in infants is rare but crucial to preventing severe complications such as recurrent cholangitis, liver fibrosis, and portal hypertension. Physicians should maintain a high index of suspicion for Caroli syndrome in cases of persistent abdominal distension, as early recognition can facilitate appropriate imaging, timely intervention, and improved patient outcomes.

## Introduction

Caroli syndrome is a rare condition, with an estimated incidence of 1 per million. It is characterized by congenital segmental dilation of the intrahepatic bile ducts, often diffuse, and is associated with hepatic fibrosis, eventually progressing to juvenile portal hypertension [1]. Although present from birth, this congenital anomaly is often not detected until adulthood. Diagnosis is usually delayed due to the clinical latency of the disease [2]. This latency, resulting from the absence of specific symptoms, often leads to its discovery only at the stage of complications, such as intrahepatic lithiasis. However, cases of portal hypertension, infections, and pancreatitis have also been reported [2].

Here, we present the case of a two-year-old infant who was diagnosed early with Caroli syndrome. We describe the clinical characteristics, diagnostic methods, and therapeutic options implemented.

## Case presentation

A one-year-and-eight-month-old infant, born to parents in a first-degree consanguineous marriage, with a history of a well-monitored twin pregnancy without complications, carried to term. The delivery was medically assisted via the upper route, with a birth weight of 2.5 kg. The child was breastfed with mixed feeding, and complementary foods were introduced at six months. Vaccinations were administered according to the National Immunization Program.

The child was admitted to the pediatric department for abdominal distension. The medical history dates back to the age of one year when the family first noticed slight abdominal distension, which was initially neglected. However, the family sought specialized care from our pediatric service due to its progressive increase.

The child does not present with jaundice or hemorrhagic syndrome, and the symptoms developed in the context of a generally preserved condition. Upon clinical examination, the child was conscious, hemodynamically and respiratory stable, and afebrile, with a weight of 15 kg (+1 SD) and a height of 96 cm (+1 SD).

Abdominal examination revealed a distended abdomen with an umbilical circumference of 56 cm and significant hepatomegaly, particularly affecting the left lobe of the liver, along with splenomegaly and collateral venous circulation. The pleuropulmonary and cardiovascular examinations showed no abnormalities. The lymphatic regions were unremarkable, and the remainder of the somatic examination was normal.

The biological workup did not reveal any significant anomalies. Similarly, plasma protein electrophoresis was normal, the bone marrow examination showed no surcharge cells, and the Guthrie test was without abnormalities (Table 1).

**Table 1:** Biological analysis showing no significant abnormalities detected

Lab parameters	Patient's normal values	Reference range
Hemoglobin	9.6 g/dL	12-16 g/dL
Mean corpuscular volume	64.4 fL	80.00-98.00 fL
Mean corpuscular hemoglobin	19.4 pg	27.00-32.00 pg
Platelets	392,000 µL	150,000-400,000/µL
Leukocytosis	11,710 µL	4,000-10,000/µL
Neutrophils	4,700 µL	1,500-7,000/µL
Lymphocytes	5,570 µL	1,000-4,000/µL
Aspartate aminotransferase	27 UI/L	5.00-34.00 UI/L
Alanine aminotransferase	23 UI/L	0.00-55.00 UI/L
Gamma-glutamyl transferase	22 UI/L	12-64 UI/L
Alkaline phosphatase	201 UI/L	<500 UI/L
Total bilirubin	2.5 mg/L	2.00-12.00 mg/L
Direct bilirubin	1.0 mg/L	0.00-5.00 mg/L
Alpha-fetoprotein	2.64 ng/mL	<7 ng/mL
Albumin	45 g/L	35-50 g/L
Prothrombin time	86%	70.00-100.00%
Sodium	143 mmol/L	138-145 mmol/L
Potassium	3.8 mmol/L	3.4-4.7 mmol/L
Total proteins	66 g/L	56-75 g/L
Calcium	94 mg/L	88-108 mg/L
Urea	0.29 g/L	0.10-0.30 g/L
Creatinine	3.42 mg/L	4.18 mg/L
Ferritin	9.21 ng/mL	4.63-204 ng/mL
Viral serologies (syphilis, Epstein-Barr virus, hepatitis A, B, C)	Negative	-
Immunoglobulin G	10.26 g/L	5.40-18.22 g/L
Immunoglobulin M	1.09 g/L	0.22-2.93 g/L
Immunoglobulin E	272 UI/mL	<60 UI/mL
Immunoglobulin A	0.9 g/L	0.63-6.45 g/L
Human immunodeficiency virus serology	Negative	-
Plasma protein electrophoresis	Normal	-
Bone marrow aspiration	No storage cells detected	-

**Figure 1:**
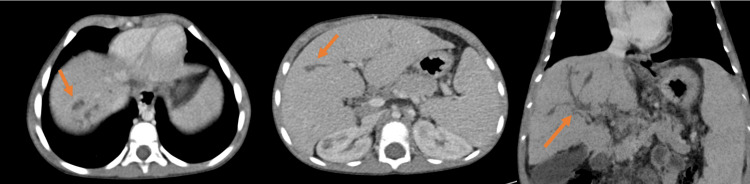
Abdominopelvic CT angiography revealing cystic dilations of the intrahepatic bile ducts in segment VII, measuring 8 mm, along with fusiform peripheral and central dilations communicating with the biliary tree, without any visible obstruction. The liver appears dysmorphic and hypertrophic CT: computed tomography

Radiological assessment shows that the abdominal X-ray does not reveal any adrenal calcifications suggestive of Wolman disease. The abdominal ultrasound shows a heterogeneous, enlarged liver, a homogeneous spleen with increased volume, no intra-abdominal mass, and no signs of portal hypertension or ascites. The abdominal CT angiogram reveals non-obstructive cystic and fusiform dilation of the bile ducts, along with a dysmorphic liver displaying signs of portal hypertension, including splenomegaly and portosystemic shunts (Figure 1).

During the upper endoscopy, no varices were observed, and renal ultrasound did not reveal polycystic kidney disease or Cacchi-Ricci disease. The clinical, biological, and radiological data suggest that Caroli syndrome is the primary diagnosis. Therefore, the patient received conservative treatment and was discharged with regular medical follow-up.

## Discussion

Caroli disease and Caroli syndrome are rare congenital conditions affecting the intrahepatic bile ducts. There is some uncertainty about whether these two forms represent distinct disorders or variations of a single disease differentiated by hepatic fibrosis [2]. Many experts believe that the two conditions are simply different stages of the same disease, as both are characterized by the dilatation of the intrahepatic biliary tree [2].

Caroli disease refers to cases where the condition is limited to ectasia or segmental dilatation of the larger intrahepatic ducts. In contrast, Caroli syndrome also involves malformations of the smaller bile ducts along with congenital hepatic fibrosis. This fibrosis can be diffuse or segmental, typically affecting one lobe of the liver, with the left lobe being more commonly involved [2].

The clinical presentation of Caroli syndrome reflects both congenital hepatic fibrosis in terms of portal hypertension and Caroli disease in terms of recurrent cholangitis and cholelithiasis [3]. Complications of Caroli disease include cholangitis, intrahepatic calculi, biliary abscess, and pancreatitis [4].

Laboratory findings in Caroli syndrome are non-specific, with possible mild transaminase elevation and blood count changes if portal hypertension is present [3]. Imaging techniques such as ultrasound, CT, magnetic resonance cholangiopancreatography, and endoscopic retrograde cholangiopancreatography aid in diagnosis by identifying communication between sacculi and bile ducts. MRI is a key non-invasive tool that confirms the diagnosis, detects associated conditions like cirrhosis and renal disease, and can reveal the characteristic "dot sign" of ductal plate malformation [5]. Doppler ultrasound helps monitor disease progression by visualizing fibrovascular bundles [6]. ERCP remains the gold standard for diagnosing fusiform biliary dilations, while histopathology confirms congenital hepatic fibrosis through bile duct ectasia and periportal fibrosis.

Given that cholangiocarcinoma develops in 7% of these patients, those with bilobar disease should undergo regular clinical monitoring via ultrasound, with biopsy if necessary [7].

In our case, the child was diagnosed at a relatively young age. During the physical examination, significant hepatomegaly with a firm consistency and splenomegaly of similar firmness raised suspicion of the condition. This initial clinical assessment prompted further investigations, including an abdominal ultrasound and a CT scan. Findings from these diagnostic tests ultimately confirmed the diagnosis.

Renal complications occur in up to 60% of cases of Caroli syndrome and include dilatation of the collecting renal tubules and kidney lesions, such as renal tubular ectasia (medullary sponge kidney, cortical cysts), lesions associated with adult recessive polycystic kidney disease and rarely, autosomal dominant polycystic kidney disease [3]. The syndrome is linked to PKHD1 gene mutations, leading to ciliary dysfunction and cyst formation. It may also be associated with other organ abnormalities, such as pulmonary fibrosis, congenital heart disease, and portal vein transformations [8].

Therapy is directed toward controlling the associated complications of cholangitis, hepatic abscesses, cholangioma, and carcinoma. Recurrent cholangitis can be managed through open surgical drainage, stent placement, or percutaneous drainage. However, such therapy is purely symptomatic [1]. In our patient, none of these complications developed during the current episode; hence, no specific therapy for these complications was required. In cases where the disease is localized to one lobe, partial lobectomy is the preferred surgical treatment. Liver transplantation remains the only curative treatment, particularly in recurrent cholangitis or malignant transformation [5]. If renal disease is present, combined liver-kidney transplantation may be necessary. Portal hypertension requires management with blood products, vasopressors, endoscopic procedures, or portosystemic shunting [9]. Due to its autosomal recessive inheritance, genetic counseling is recommended for affected families [10].

## Conclusions

The early diagnosis of Caroli syndrome in a one-year-and-eight-month-old infant is rare but crucial for preventing severe complications. Unlike the more common late discovery in adulthood, early detection allows timely medical intervention. In this case, the infant presented with abdominal distension and hepatosplenomegaly, prompting further investigation. Imaging studies, including an abdominal ultrasound and CT angiography, revealed segmental dilation of the intrahepatic bile ducts, characteristic of Caroli syndrome. Early diagnosis enabled close monitoring and the implementation of management strategies to prevent complications such as intrahepatic lithiasis and recurrent infections. This case highlights the importance of considering congenital biliary anomalies in young patients with unexplained hepatobiliary symptoms, as early detection significantly improves prognosis and quality of life.
